# Benzoate of Soda in Diphtheria

**Published:** 1880-01

**Authors:** 


### BENZOATE OF SODA IN DIPHTHERIA.

Dr. Letzrich, of Berlin, has been studying the effect of the above remedy in diphtheria. It has been shown, he alleges, by the experiments of Graham, that this remedy when introduced into the system in sufficient quantity will put a stop to the "vegetation of the specific poison." The amount necessary for this purpose is determined by the weight of the body. In this manner, accordingly, the dose for children and adults is regulated, and it is claimed by him, that up to the present time, there is no other remedy that exercises so rapid, continuous and therapeutic an effect upon the development and cause of the diphtheritic process as benzoate of soda. The dose for children between one and three years old is given as seven to eight grammes (100 to 120 grains), dissolved in three and one-half ounces of the vehicle, the whole amount being given in the course of the day, in half to one tablespoonful doses.

For children between three and seven years of age, eight to ten grammes (2 to 2½ drachms) are given in the same way.

Those over seven years old take ten to fifteen grammes, and adults fifteen to twenty-five grammes daily—dissolved in a suitable vehicle.

The diphtheritic membrane was treated with benzoate of soda or powder being sprinkled on or applied through a glass tube or quill. There is no slough formed, and thereby the danger is averted of its acting as a firm covering under which an energetic growth and development of the organisms may take place.

The insufflation was made every three hours in severe cases, in the milder form two or three times daily. With older children a simple solution of the salt was used as a gargle.

The author cites the following case as a typical illustration of the way the medicine acts upon the general infection, the effects being quite uniformly noticed after twenty-four to thirty-six hours. W. L., eight years old; treatment began June 19, the second day of the disease; June 19, evening, 106.3° Fahr., pulse 136; 20, evening, 102.2° Fahr., pulse 124; 21, morning, 101.6° Fahr., pulse 114; 21, evening, 100.4° Fahr., pulse 112; 22, morning, 99.5° Fahr., pulse 104; 22, evening, 98.6° Fahr., pulse 104; 23, temp. normal, pulse normal.

In the above case the membrane on the tonsils was very extensive and was powdered. On the 2d day of the disease it became circumscribed, thinner and somewhat more transparent, and on the fifth it had nearly disappeared. The medicine was continued for a few days after this date, but at longer intervals, and the small exudation spots were powdered twice daily, until the last remaining portion had completely disappeared, on the 8th day of the disease.

The records of many other children equally severely affected, and of different ages, gave nearly the same results as the above, and the effects of the medicine were always the same. The author recommends this remedy highly in gastric and intestinal disease, particularly of infants, and states that at times the results are surprising in the latter cases. He recommends it likewise in catarrh of the bladder, and firmly believes in the statement of Klebs, that it is to be recommended in all diseases which originate by infection.—*Boston Medical and Surgical Journal.*

